# Lipidated Peptidomimetic Ligand-Functionalized HER2 Targeted Liposome as Nano-Carrier Designed for Doxorubicin Delivery in Cancer Therapy

**DOI:** 10.3390/ph14030221

**Published:** 2021-03-06

**Authors:** Himgauri Naik, Jafrin Jobayer Sonju, Sitanshu Singh, Ioulia Chatzistamou, Leeza Shrestha, Ted Gauthier, Seetharama Jois

**Affiliations:** 1 School of Basic Pharmaceutical and Toxicological Sciences, College of Pharmacy, University of Louisiana at Monroe, Monroe, LA 71201, USA; himgaurinaik@gmail.com (H.N.); sonjujj@warhawks.ulm.edu (J.J.S.); singhss@warhawks.ulm.edu (S.S.); leezashr14@gmail.com (L.S.); 2 Department of Pathology, Microbiology & Immunology (PMI), School of Medicine, USC, SC 6439 Garners Ferry Rd, Columbia, SC 29208, USA; Ioulia.Chatzistamou@uscmed.sc.edu; 3 Biotechnology Laboratory, LSU AgCenter, Louisiana State University, Baton Rouge, LA 70803, USA; TGauthier@agcenter.lsu.edu

**Keywords:** epidermal growth factor receptor (EGFR), liposome, doxorubicin, HER2, NSCLC

## Abstract

The therapeutic index of chemotherapeutic agents can be improved by the use of nano-carrier-mediated chemotherapeutic delivery. Ligand-targeted drug delivery can be used to achieve selective and specific delivery of chemotherapeutic agents to cancer cells. In this study, we prepared a peptidomimetic conjugate (SA-5)-tagged doxorubicin (Dox) incorporated liposome (LP) formulation (SA-5-Dox-LP) to evaluate the targeted delivery potential of SA-5 in human epidermal growth factor receptor-2 (HER2) overexpressed non-small-cell lung cancer (NSCLC) and breast cancer cell lines. The liposome was prepared using thin lipid film hydration and was characterized for particle size, encapsulation efficiency, cell viability, and targeted cellular uptake. In vivo evaluation of the liposomal formulation was performed in a mice model of NSCLC. The cell viability studies revealed that targeted SA-5-Dox-LP showed better antiproliferative activity than non-targeted Dox liposomes (Dox-LP). HER2-targeted liposome delivery showed selective cellular uptake compared to non-targeted liposomes on cancer cells. In vitro drug release studies indicated that Dox was released slowly from the formulations over 24 h, and there was no difference in Dox release between Dox-LP formulation and SA-5-Dox-LP formulation. In vivo studies in an NSCLC model of mice indicated that SA-5-Dox-LP could reduce the lung tumors significantly compared to vehicle control and Dox. In conclusion, this study demonstrated that the SA-5-Dox-LP liposome has the potential to increase therapeutic efficiency and targeted delivery of Dox in HER2 overexpressing cancer.

## 1. Introduction

The major drawback associated with currently available chemotherapeutics is the lack of targeted delivery toward cancer cells. This non-selective effect leads to increased toxicity to healthy cells [1,2]. The therapeutic index of chemotherapeutic agents can be improved by the use of nano-carrier-mediated chemotherapeutic delivery. Nano-carrier-mediated chemotherapeutic delivery has been shown to maximize the therapeutic index and decrease toxicity toward normal cells [2]. The use of nano-carrier liposomes is known to be a successful strategy for increasing the delivery of anti-cancer drugs to tumors [3]. Polyethylene glycol (PEGylated)-incorporated liposomes are stealth liposomes that offer improved half-life of a formulation in vivo. Nanoparticles with a diameter between 20–200 nm are suitable for drug delivery to cancer cells as they can easily penetrate leaky tumor vasculature and achieve longer retention times; this effect is known as enhanced penetration and retention (EPR) [4,5].

Ligand-targeted drug delivery can be used to achieve selective and specific delivery of chemotherapeutic agents to cancer cells. Targeted delivery of anti-cancer agents can be achieved by incorporation or conjugation of specific ligands such as monoclonal antibodies, peptides, or proteins with the nano-carrier [6,7]. Cancer cells are known to overexpress specific receptors [8]. Those overexpressed receptors can be targeted by a suitable ligand that has a specificity and affinity toward a receptor. Popular ligand-targeted therapies available employ drug-antibody conjugates; ligand-functionalized liposomes, and radiolabeled immunotherapeutics [3,9]. The use of ligand-targeted liposomes is known to increase drug delivery of chemotherapeutics to cancer cells and reduce toxicity to normal cells [7,10]. In comparison with free chemotherapeutics, liposomal chemotherapeutics are known to achieve longer bioactivity and tumor localization and less toxicity. Examples of clinically approved liposome formulations are Doxil^®^, Onivyde^®^, and Marqibo^®^ for cancer treatment, and several others are in clinical trials [11,12].

**Figure 1:**
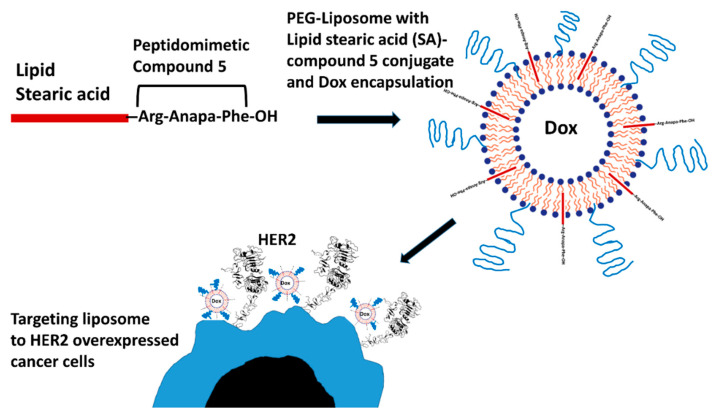
Schematic diagram of Dox-encapsulated, a lipid–peptide conjugated tagged liposome. Compound 5 that targets HER2 protein is conjugated with lipid stearic acid (SA-5). The conjugate was incorporated into a doxorubicin-encapsulated liposome (SA-5-Dox-LP). The lipid part of the conjugate is incorporated into the liposome with compound 5 on the liposome surface. Compound 5 is specific for HER2 extracellular domain IV and binds to HER2 along with the liposome.

Different choices of ligands, such as antibodies, peptide, protein, folic acid, transferrin, and albumin, are available for targeted delivery [10]. Despite the many advances in innovative drug delivery systems, targeted delivery of chemotherapeutics to cancer cells remains a major clinical challenge [13]. The therapeutic efficiency of chemotherapeutics agents can be improved by encapsulating drugs into nanoparticles and conjugating nanoparticles with targeting ligand, which binds to tumor cells specifically. Liposomes are spherical vesicles with at least one bilayer. Therefore, attachment of specific ligands that target liposomes to tumor cells is essential to improve tumor-targeted therapy [6,9]. Among them, peptides are a good choice because of their lower immunogenicity, suitable size, and ease of chemical modification. Studies have indicated that peptide-targeted liposomal delivery of doxorubicin, paclitaxel, and gemcitabine provided improved therapeutic efficacy and decreased toxicity in xenograft animal models [14]. Here we describe the application of novel lipidized peptidomimetic conjugate SA-5 as a ligand (Figure 1) for the preparation of liposomal chemotherapeutics targeted toward human epidermal growth factor receptor -2 (HER2) overexpressed cancer cells. In our previous study, we showed that SA-5 exhibited better selectivity for HER2 (EGFR) overexpressed cancer cells and indicated affinity to HER2 protein [15]. The objective of this work was to evaluate the potential of SA-5 as a ligand for HER2 overexpressing cancer targeting by liposomal chemotherapeutics carrier. 

Our idea of the design for SA-5 was to use compound 5, which is known to bind to HER2 protein extracellular domain, as the ligand and conjugate stearic acid (SA) to it so that it can be incorporated into the liposome [15,16]. The ligand-incorporated liposome can be encapsulated with Dox or other chemotherapeutic agents to target the chemotherapeutic drug to HER2 protein that is overexpressed on cancer cells (Figure 1). The members of the EGFR family of proteins are known to be overexpressed in different types of cancers such as breast, lung, ovarian, and colon [8]. HER2 is known to be overexpressed/altered in 30% of breast cancer and 4–25% of lung cancer [17,18]. Compound 5 is known to be a dimerization inhibitor of HER2 with other members of EGFR [19]. Dimers of EGFR:HER2 and HER2:HER3 are known to drive the oncogenic signaling in breast and non-small cell lung cancers [20,21]. The peptidomimetic part of the molecule can target HER2 overexpressed cancer cells and inhibit the dimerization of HER2 with other receptors hence inhibit the downstream signaling for cell growth in cancer cells. Dox incorporated in the liposome is a chemotherapeutic agent that is known to kill cells by targeting DNA and topoisomerase enzyme [22]. Thus, the prepared ligand-targeted liposome can be used to treat breast and lung cancers that are HER2 positive. The liposome we prepared will have two effects, inhibition of EGFR dimerization hence inhibition of growth of cancer cells, and Dox will have an anti-cancer effect in addition to this.

Liposomal Dox formulations Doxil^®^ and Caelyx^®^ were approved by the United States Food and Drug Administration Agency (FDA) for the treatment of AIDS-related Kaposi’s sarcoma and ovarian cancer [2,9,11,12]. Doxil^®^ with other conventional therapies has been tested for NSCLC; the results from Phase I and Phase II testing were promising [23]. In the present study, we formulated an SA-5-functionalized Dox liposome (SA-5-Dox-LP) to achieve targeted delivery of the anti-cancer agent Dox toward HER2 overexpressed cancer cells. The use of free Dox is limited due to its several side effects, such as cardiotoxicity, hair loss, and nephrotoxicity. Dox has been encapsulated in liposomes to decrease the side effects associated with free Dox treatment. A remote drug loading approach is used for achieving high yield in Dox encapsulation in liposomes [24,25]. This strategy involves the use of a pH gradient or an ion gradient capable of generating a pH gradient to incorporate the drug into the preformed liposome. The SA-5-Dox-LP formulation was further assessed for its characteristics such as particle size, encapsulation efficiency, serum stability, antiproliferative activity, and cellular uptake. The liposomal formulation was evaluated for its targeting effect in an in vivo model of non-small cell lung cancer. The formulation was assessed for its effectiveness in reducing the tumor growth in NSCLC in mice compared to the control.

## 2. Results

### 2.1. Characteristics of Dox-Encapsulating SA-5 Peptidomimetic Liposomes 

**Figure 2:**
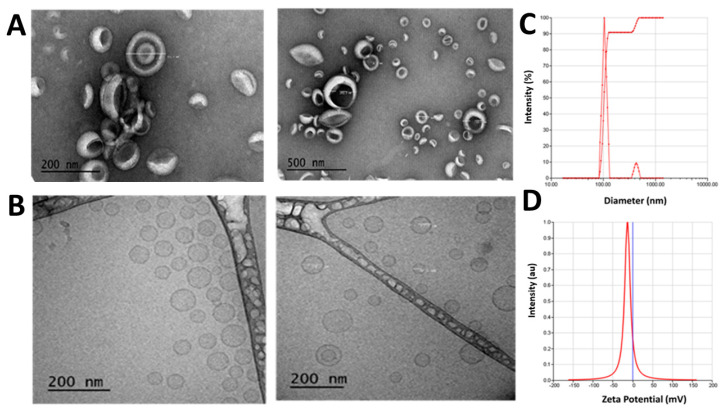
(**A**) TEM images in different magnifications, (**B**) Cryo-TEM images (**C**) size distribution graph, and (**D**) zeta potential graph of SA-5-Dox-LP.

Liposome formulations SA-5-Dox-LP and Dox-LP were prepared by thin-film hydration using lipids dipalmitoylphosphatidylcholine (DPPC), cholesterol, and poly(ethylene glycol) distearoylphosphatidylethanolamine (PEG-DSPE2000). The average particle size for SA-5-Dox-LP and Dox-LP was found to be 107.19 nm and 105.23 nm, respectively (Figure 2A–C and Supplementary Materials, Figure S1). The particle size of liposomes is within an optimal size range for retention of nano-carrier into the tumor tissue (Figure 2C). The polydispersity index (PDI) for liposomes was around 0.17 for liposome preparations, suggesting the homogeneity of liposomes [26]. Zeta potential for Dox-LP was −4.18 mV, and for SA-5-Dox-LP was −13.38 mV (Figure 2D). To ensure that the particle size is within the narrow range and spherical shape of liposomes, transmission electron microscopy (TEM) images of liposomes were obtained (Figure 2A and Supplementary Materials, Figure S1). Cryo-TEM images were also analyzed, which showed unilamellar liposome vesicles of SA-5-Dox-LP (Figure 2B). Analysis of the images indicated that most of the liposomes were spherical, <150 nm in size, and unilamellar. The encapsulation efficiency of Dox in Dox-LP and SA-5-Dox-LP was around 85%. Plain LP showed a smaller particle size and zeta potential compared to other liposome formulations (Table 1).

### 2.2. Stability of Liposomes

**Figure 3:**
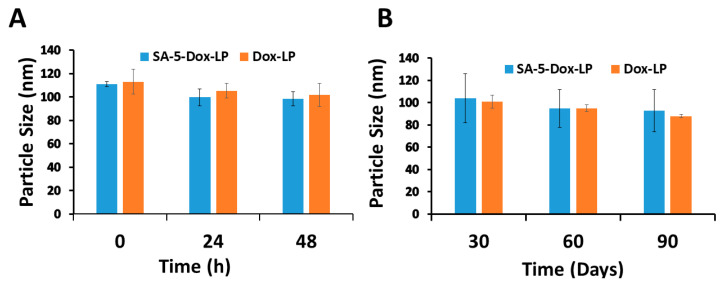
(**A**) In vitro serum stability of SA-5-Dox-LP and Dox-LP was measured by particle size evaluation of liposomes. (**B**) Storage condition stability of SA-5-Dox-LP and Dox-LP at 4 °C was measured by particle size evaluation of liposomes. Data represented as mean ± standard deviation. LP-liposome.

In vitro serum stability of liposomes SA-5-Dox-LP, Dox-LP were investigated using a human serum at 37 °C and 0, 24, and 48 h points. The particle size of liposomes was measured as a marker of the stability of liposomes. The overall particle size of the liposomes incubated in human serum for different durations was relatively constant, as seen in Figure 3A. Next, the stability of liposomes at 4 °C storage conditions was also examined for 90 days. The relative particle size of SA-5-Dox-LP and Dox-LP showed slight changes during this testing period, indicating the stability of liposome for 90 days at 4 °C, as seen in Figure 3B.

### 2.3. Antiproliferative Activity

The antiproliferative activity of liposomes was evaluated in HER2 overexpressed breast and lung cancer cell lines BT-474, A549, Calu-3, and MCF-7 cells that do not overexpress the HER2 protein. BT-474 is a HER2 positive breast cancer cell line; Calu-3 and A549 are HER2 positive lung cancer cell lines. MCF-7 is a HER2 negative breast cancer cell line. As a control, a non-cancerous human lung fibroblast cell line (HLF) was used. Targeted SA-5-Dox-LP formulations showed antiproliferative activity in a dose-dependent manner on HER2 overexpressing cancer cells. As reported in the literature, free Dox showed the highest antiproliferative activity compared to the liposomal formulations. IC_50_ values calculated indicated that SA-5-Dox-LP has relatively lower IC_50_ values compared to Dox-LP in BT-474 and Calu-3 HER2 expressing cell lines. (Table 2). IC_50_ values of A549 cells are similar for SA-5-Dox-LP and Dox-LP. This may be due to different levels of HER2 expression in these cell lines. Calu-3 and BT-474 have very high expression of HER2 protein compared to A549 [27].

The more potent antiproliferative effect by SA-5-Dox-LP formulation compared to Dox-LP could be attributed to the combined effect of SA-5 and Dox antiproliferative activity. On the other hand, on the MCF-7 cell line, which does not express HER2, targeted SA-5-Dox-LP formulation showed less antiproliferative activity, with an IC_50_ value of 2.2 µM compared to free Dox. Furthermore, free Dox showed lower cell viability (Table 2). The results showed that the inclusion of SA-5 as a ligand also played a role in the anti-tumor effect of SA-5-Dox-LP formulation since SA-5 has antiproliferative activity as described in our earlier report [15]. Additionally, SA-5-Dox-LP showed lower cellular toxicity toward human lung fibroblast cells (HLFs), indicating the non-selective nature of ligand-functionalized liposome for non-cancerous cells that do not overexpress the HER2 protein.

### 2.4. Time-Dependent In Vitro Cellular Uptake

**Figure 4:**
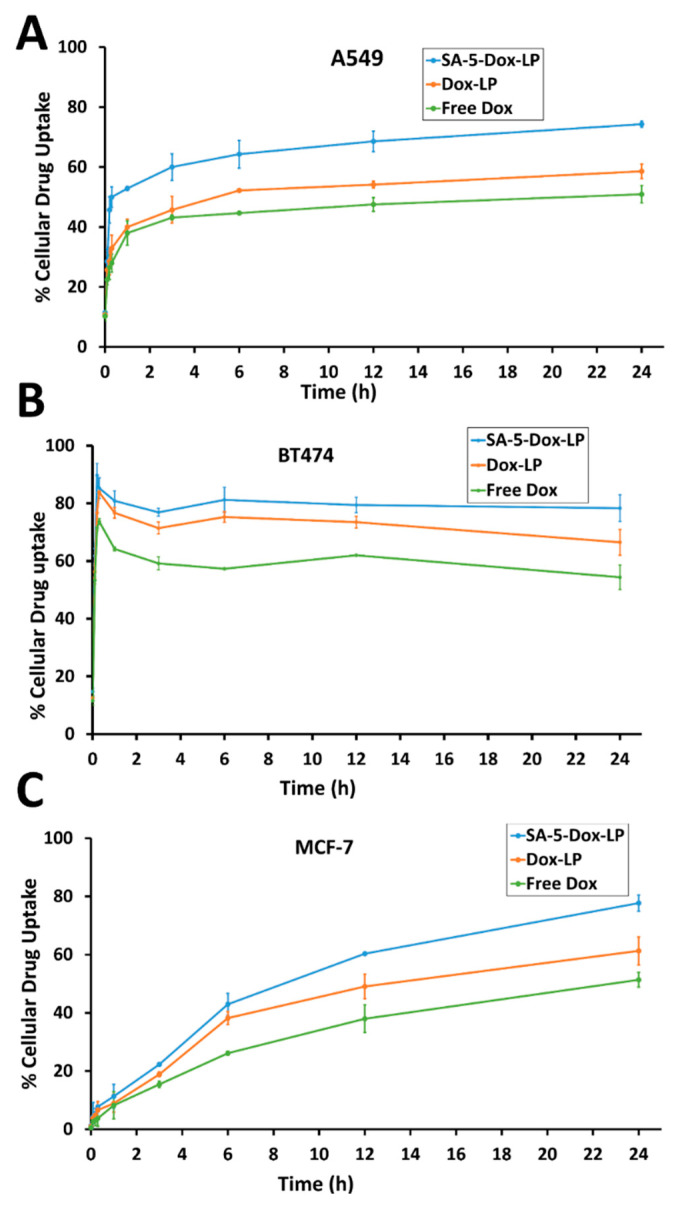
(**A**) Cellular uptake of doxorubicin by SA-5-Dox-LP, Dox-LP, and free Dox in BT-474 (HER2 + ve) cell line at 0 to 24 h. Data represented as mean ± standard deviation. LP-liposome (**B**) Cellular uptake of doxorubicin by SA-5-Dox-LP, Dox-LP, and free Dox in A549 (HER2 + ve) cell line at 0 to 24 h. Data represented as mean ± standard deviation. LP-liposome. (**C**) Cellular uptake of doxorubicin by SA-5-Dox-LP, Dox-LP, and free Dox in MCF7 (HER2-ve) cell line at 0 to 24 h. Data represented as mean ± standard deviation. LP-liposome.

To evaluate the in vitro cellular uptake of peptidomimetic liposomes loaded with Dox, the fluorescence intensity of Dox was measured (Figure 4A–C). Quantitative analysis of cellular uptake in HER2-positive A549 and BT-474 cell lines showed greater fluorescence intensity of SA-5-Dox-LP compared to that of free Dox and Dox-LP. Stronger fluorescence by SA-5-Dox-LP indicates that SA-5 could facilitate greater cellular uptake of liposome formulations. In BT-474 cell lines, cellular uptake studies indicated a biphasic effect with a saturation of uptake from 0 to 30 min and then a slight increase in cellular uptake after 30 min to saturation within 2 h. In A549 cells, there was a steep increase in cellular uptake for 30 min, and then the saturation effect was observed. However, in MCF-7 cell lines, there was a continuous increase in cellular uptake of SA-5-Dox-LP in a linear fashion. In all three cell lines, free Dox showed decreased cellular uptake after initial uptake for 30 min. Overall, fluorescence intensity increased with extended incubation time, which shows that cellular uptake of liposome formulation occurred in a time-dependent manner. Standards curves were generated using the known concentration of Dox (Supplementary Materials, Figure S2).

The total protein concentration of the three different cell lines A549, BT474, and MCF-7 was evaluated to determine the living cells up to 24 h of SA-5-Dox-LP treatment. The total protein concentration was similar up to 12 h of treatment but slightly reduced at 24 h of treatment in all three cell lines (Supplementary Materials, Figure S3).

### 2.5. In Vitro Fluorescence Microscopy Studies

**Figure 5:**
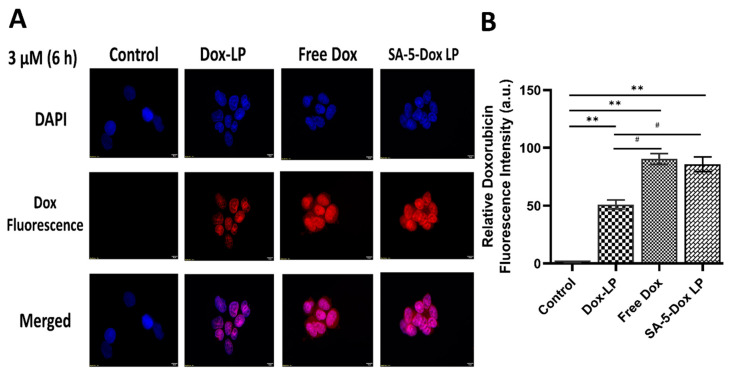
(**A**) Fluorescence images of cellular uptake studies of SA-5-Dox-LP, Dox-LP, and free Dox in BT-474 cells. Cells were incubated with different formulations for 6 h and fixed after washing. Fluorescence from doxorubicin was imaged along with nuclear stain 4′,6-diamidino-2-phenylindole (DAPI). The figure shows doxorubicin fluorescence and DAPI fluorescence, and merged images. The merged image indicates that in 6 h, SA-5-Dox-LP formulation was taken up by cells and entered the nucleus. Clusters of cells are shown. LP-liposome. Magnification 60×. (**B**) Quantification of relative Dox fluorescence of the images of cellular uptake studies of SA-5-Dox-LP, Dox-LP, and free Dox in BT-474 cells. Sample size n = 3. Statistical analysis was done using the two-tailed student t-test. # *p* < 0.05, ** *p* < 0.01. Mean ± SD.

Fluorescence microscopy images displaying BT-474 cells treated with different Dox liposome formulations are shown in Figure 5A. These indicate that intracellular localization of targeted SA-5-Dox-LP after 6 h is greater than in Dox-LP in BT-474 cells. In this study, targeted ligand peptide SA-5 may facilitate more uptake of Dox liposome formulation inside cells compared to non-targeted Dox liposome and free Dox. The results indicate that SA-5 on the liposome helps to bind to HER2 expressed on BT-474 cells, and then Dox is released relatively slowly over 6 h in cells. For comparison, similar studies were carried out in MCF-7 cell lines in supporting information (Supplementary Materials, Figure S4). Quantitative analysis of the images also reveals that the SA-5-Dox-LP had more Dox fluorescence compared to Dox-LP in BT-474 cells (Figure 5B).

### 2.6. In Vitro Release of Dox from Liposomes Formulations

**Figure 6:**
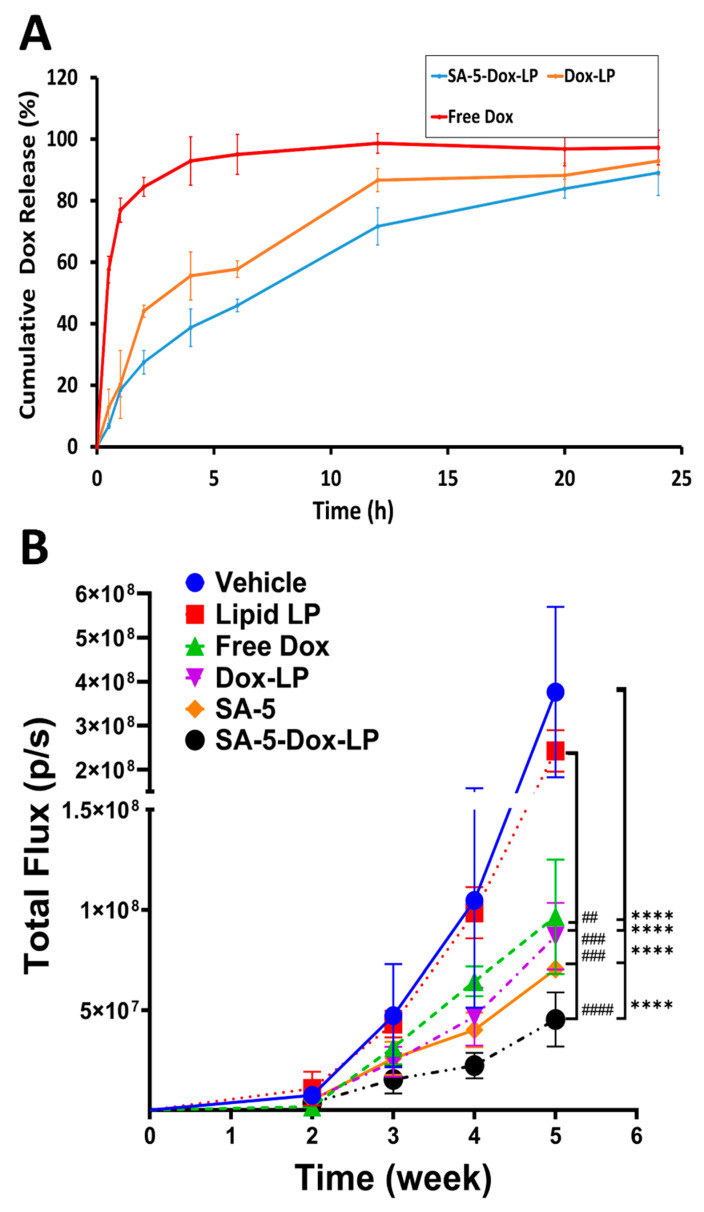
(**A**) In vitro drug release from SA-5-Dox-LP and Dox-LP at pH 7.5. Results were from triplicate experiments. Dox release was monitored by measuring the fluorescence of doxorubicin and expressed as a percentage calculated as described in the text. LP-liposome. Each data represents mean ± S.D. (**B**) Anti-tumorigenic activity of SA-5, SA-5-Dox-LP, Dox-LP, free doxorubicin, lipid liposome: SA-5, 6 mg/kg and SA-5-Dox-LP formulation group showed significantly delayed tumor growth as compared to control vehicle and lipid, liposome group. Each data represents mean ± S.D. Statistical analysis: two-way analysis of variance (ANOVA) with Tukey’s test for multiple comparisons. ## *p* < 0.01, ### *p* < 0.001, #### *p* < 0.0001, **** *p* < 0.0001.

The results of the in vitro release of Dox from SA-5-Dox-LP, Dox-LP formulations, and free Dox in phosphate buffered saline (PBS) at pH 7.5 are shown in Figure 6A. The free Dox release curve refers to Dox that was inside the dialysis bag and was released into the dialysis medium. Dox was gradually released from the SA-5-Dox-LP formulation from around 40% at 4 h and reached to around 80% in 12 h. In Dox-LP formulations, the Dox release was more than 80% at 12 h, and after that, there was a steady release of Dox from the liposomal formulation. For SA-5-Dox-LP and Dox-LP formulations groups, the release of Dox is similar at pH 7.5 from 2 to 24 h, indicating less effect on Dox release by the incorporation of the peptidomimetic-lipid conjugate in the liposome. However, the Dox release reached more than 80% within 2 h in the release medium from the free Dox solution. We have also carried out the Dox and its conjugate release in the formulation of liposome without the addition of tween in PBS for release studies. There is a slight difference in the release of Dox-LP and SA-5-Dox-LP with and without tween (Figure S5). However, there was no change in free Dox release with and without tween in PBS. The release of SA-5-Dox in SA-5-Dox-LP in 24 h was the same with and without tween in PBS.

### 2.7. In Vivo Activity

To investigate the in vivo anti-tumor effect of functionalized targeted liposomes, mice with lung tumors were injected with different liposomal formulations twice a week for three weeks. A selected dose of SA-5 for in vivo study was 6 mg/kg twice a week. Dose selection based on our previous data on compound 5 evaluated with different doses on xenograft tumor model and dosing of 6 mg/kg was chosen based on efficacy and enzymatic degradation susceptibility in vivo condition [28]. Furthermore, a selected dose for Dox was 4 mg/kg. This dose selection based on studies reported previously [29,30]. Tumor growth was monitored once a week by bioluminescence imaging of the tumor tissue (A549 Red-FLuc NSCLC cells). The photon flux from tumors is directly proportional to the number of light-emitting cells that express luciferase, and the signal can be measured to monitor tumor growth and development [31]. Results showed that during treatment, the tumor size in the vehicle control group without any treatment continue an increase in tumor growth, while treatment groups showed a delay in tumor growth. Formulations SA-5 and SA-5-Dox-LP indicated a significant reduction in tumor growth compared to free Dox, Dox-LP, and control, lipid liposome (Figure 6B, Supplementary Materials, Figure S6). Statistical analysis (unpaired t-test) indicated that for weeks 4 and 5, there was a significance difference between vehicle control, SA-5, and SA-5-Dox-LP (* *p* < 0.05, ** *p* < 0.01). Furthermore, a two-way analysis of variance (ANOVA) followed by Tukey’s multiple comparisons test was used to compare the statistical difference between groups. For week 5, there was a significance difference between vehicle control and Free-Dox, Dox-LP, SA-5, and SA-5-Dox-LP (**** *p* < 0.0001). Also, there was significance difference between Lipid-LP and Free-Dox (## *p* < 0.01), Dox-LP (### *p* < 0.001), SA-5 (### *p* < 0.001), and SA-5-Dox-LP (#### *p* < 0.0001) at week 5 (Figure 6B).

### 2.8. Histopathology of Tissues and Assessment of Toxicity

**Figure 7:**
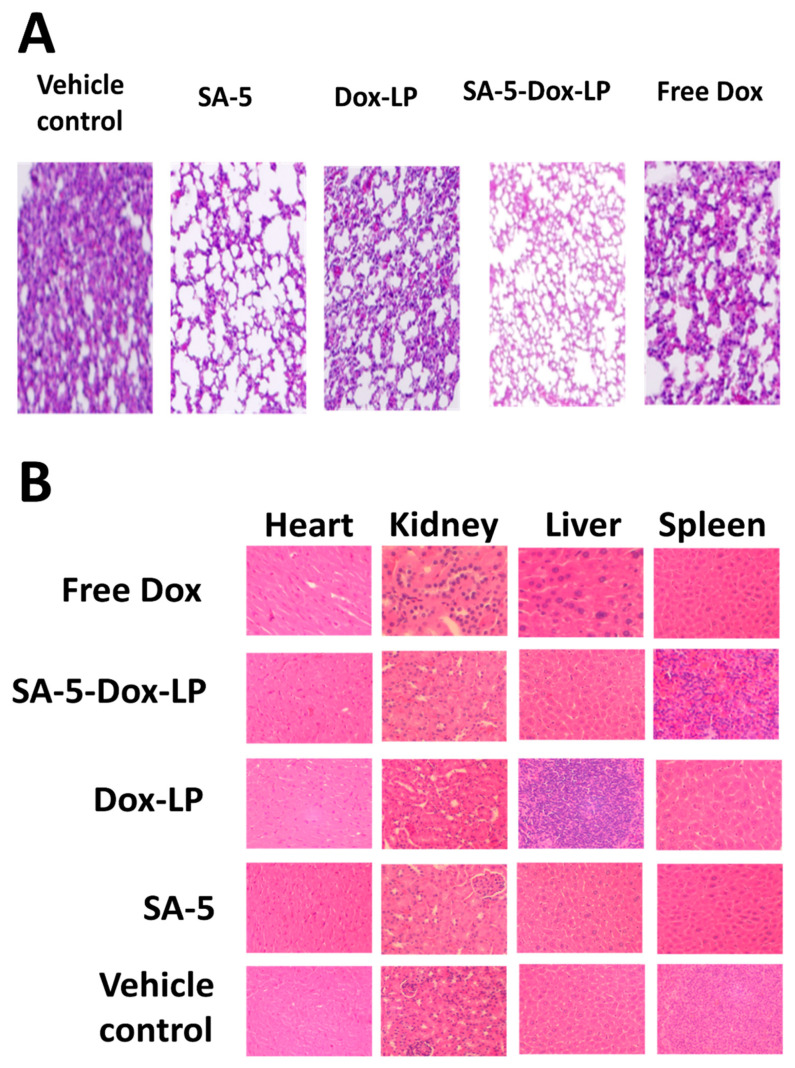
Hematoxylin and eosin (H&E) staining analysis of major organs after treatment. (**A**) SA-5-Dox-LP and SA-5 alone treated lung tumor slides exhibited normal lung morphology features, while free Dox and doxorubicin liposome treated lung tumor slides show the presence of tumor nest in the lung region. (**B**) Tissue sections of heart, kidney, liver, and spleen. General morphology indicated that there was no toxicity to major organs at the therapeutic concentration of SA-5-Dox-LP.

Tumor sections of the lungs and tissue section of organs (liver, kidney, and heart) of mice that were used in the anti-tumor activity study were analyzed for signs of cancer as well as the toxicity of the administered formulations at the dose described in the section above. In the control group treated only with a vehicle as well as in the mice group treated with free Dox, several cancer foci were seen in the lung parenchyma, while in the group treated with Dox-LP, only one small cancer focus was present. In the group treated with SA-5-Dox-LP, no cancer was present, while in the same specimen, sparse mild peribronchial inflammation was seen. The SA-5 peptide, as well as the lipid liposome, treated group did not show any histological findings in the lungs (Figure 7A). Furthermore, histological analysis of the liver showed mild periportal and perivenular inflammatory aggregates in the vehicle, the free Dox, and the SA-5 groups. All histological sections of the heart, kidney, and spleen showed normal architecture and cytology without any signs of toxicity (Figure 7B).

### 2.9. Inhibition of EGFR Heterodimerization In Vivo

**Figure 8:**
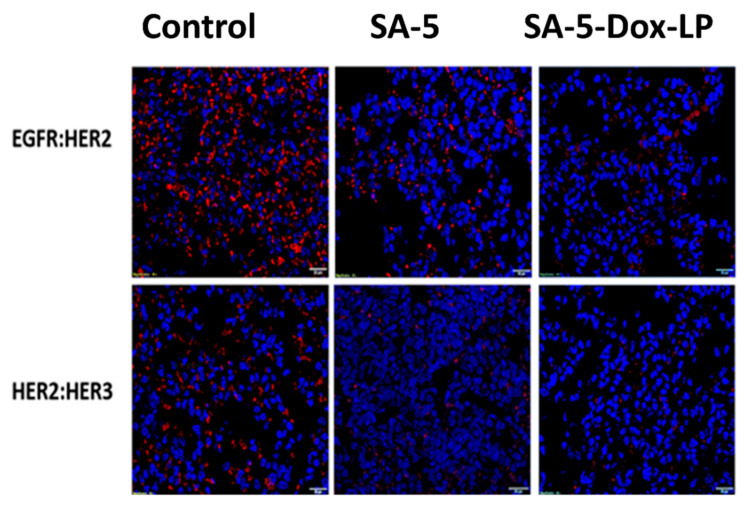
Protein-protein interaction inhibition of EGFR: HER2 and HER2:HER3 in tissue samples of experimental lung cancer model using PLA assay. Control without any compound treatment. Red fluorescent dots represent protein-protein interaction of EGFR: HER2, HER2:HER3. Tissue samples from the experimental lung cancer model treated with SA-5. Tissue samples from the experimental lung cancer model treated with SA-5-Dox-LP.

Compound 5 and lipid conjugate of 5 (SA-5) were known to bind to the HER2 protein and inhibit EGFR dimerization in cancer cell lines [15,19]. SA-5 and SA-5-Dox-LP were evaluated for their ability to inhibit EGFR heterodimerization (protein-protein interactions) in vivo sample tissues of the lungs of mice treated with these formulations. Proximity ligation assay (PLA) assay was performed to evaluate inhibition of EGFR: HER2 and HER2-HER3 heterodimerization. Samples of vehicle control groups in the tumor section of mice exhibited red fluorescent dots, suggesting the occurrence of EGFR heterodimerization, as seen in Figure 8. However, red fluorescent dots significantly decreased on the tumor section samples of SA-5 and SA-5-Dox-LP treatment groups, as seen in Figure 8 of the top panel and bottom panel. Overall, results suggested that SA-5 and SA-5-Dox-LP were able to successfully inhibit EGFR heterodimerization in vivo.

## 3. Discussion

To continue our efforts on targeted therapy, we used a HER2-binding peptidomimetic that was conjugated to a lipid to design liposomes that can be targeted to HER2/EGFR overexpressed cancer. Liposomal delivery of drugs has been used for cancer treatment to reduce the toxicity of chemotherapeutic agents. The aim of this study is to assess the ligand-targeting potential of SA-5 conjugate toward HER2-overexpressing cancer cells for the development of tumor-targeted liposomal Dox formulations. HER2 overexpression is found in some cases of NSCLC [32,33]. Non-small cell lung cancer (NSCLC) is one of the leading causes of cancer death worldwide, and chemotherapy is a widely used approach for its treatment either alone or in combination with other forms of therapy. However, the main disadvantages of conventional chemotherapy are lack of selectivity and multidrug resistance [34]. To decrease the severe toxicities of conventional chemotherapeutics on normal tissues, nanocarriers such as ligand-targeted liposomes can be used for site-specific delivery of chemotherapeutics [35,36]. Ligand-targeted therapeutic agents such as ligand-targeted liposomes showed promising results in overcoming these limitations. Ligand-targeted liposomes combine the advantages of passive targeting (liposomes) and active tumor-specific targeting (ligand) functions [10,14]. Therefore, we believe the introduction of a dual functionalized peptide in the Dox liposome formulation (SA-5 to bind to HER2 protein overexpressed in cancer and inhibit dimerization, Dox-encapsulated will act as anti-cancer agent) could enhance its therapeutic efficiency in the lung tumor model due to the synergistic effect of SA-5 and Dox.

NSCLC cell line A549 is known to overexpress HER2 and sensitive to Dox [37] treatment. Hence, using a HER2-targeted liposome, Dox was chosen for preparing liposomes for active targeted therapy for NSCLC. PEGylated Dox liposomal formulation is used in combination therapy for breast cancer, and several studies are conducted to evaluate the Dox formulations in HER2 positive breast cancer in a model system [38,39]. SA-5-Dox-LP prepared by thin-film hydration showed particle sizes around 107.19 nm (Table 1), which is suitable to penetrate leaky vasculature and enhance retention time in tumor tissue. Modification of a liposome with SA-5 does not affect the particle size of liposome formulations. Furthermore, liposome formulations were able to encapsulate Dox effectively (Table 1). Surface modification of liposomes with a polymer such as PEG is known to provide stabilization to a liposome surface and slow down liposome recognition by opsonins and, hence, clearance. Thus, PEG-modified liposomes have a relatively long plasma half-life [40]. Liposomes made up of DPPC and DSPE-PEG at a particular molar ratio are found to release nearly 45% of Dox within 20 sec after exposure to 42 °C temperature [41]. Initial particle size analysis indicated that the liposomes obtained have a particle size of 107.19 nm. Particle size is particularly important for the in vivo delivery of drugs, especially the delivery of drugs to lung tissue. Whether liposomes are coated with polymers (stealth component) or not, if the particle has a hydrodynamic diameter > 200 nm, it exhibits a faster rate of intravascular clearance (splenic and hepatic sequestration) [42]. The particle size is also important for the accumulation of liposomes in the tumor that is facilitated by highly permeable tumor blood vessels [43]. In general, the accepted particle size for long circulation time in vivo and tumor accumulation is from 100 to 300 nm, depending on the surface charge [44]. The size of the liposomes is very important for lung cancer treatment. Particles with a size of 200 nm or less can stay in the lungs for a longer period and, hence, release more of the drug from the liposome [45]. Higher ligand density on the liposome surface increases the affinity of ligand binding [46]. PEG-DSPE concentration in the formulation is less than 3% compared to targeting ligand SA-5, which is around 7.5%, providing enough drug density in the outer layer for the targeted effect. The in vitro results also suggested that the SA-5-Dox-LP acting as a vehicle for targeted delivery of Dox. The lipid portion of SA-5 is in the lipid bilayer of the liposome whereas the peptide part is exposed on the surface of liposome providing the targeting effect to bind to EGFR dimers on cancer cells.

From the physicochemical analysis, we found that the incorporation of SA-5 in the liposome layer increased the size of liposomes compared to Plain LP. However, the zeta potential decreased by the incorporation of the SA-5 in the layer of the liposomal membrane compared to the Plain LP and Dox-LP (Table 1 and Supplementary Materials, Figure S1). The incorporation of bulky SA-5 ligand increased the size of the liposome diameter because of the partial buried Dox-5 conjugate molecule in the liposome bilayer. Similar results were also reported in increasing liposome size upon the incorporation of lipidated ligand to the liposome [47]. Liposome zeta potential depends on the compounds bound on its surface [48]. SA-5 may be contributing to the generation of charge on the overall charge of the liposomes.

Our liposome formulations showed stability by maintaining a stable particle size in the presence of human serum (Figure 3). The liposome can bind to serum proteins during circulation; therefore, the aggregation behavior of the liposome in fetal bovine serum (FBS) can reflect the stability of the liposome under in vivo conditions. The recommended storage temperature for marketed Dox liposome formulations is 4 °C. To evaluate the stability of the liposomes we prepared, we measured their particle size as an indicator of stability. In serum, SA-5-Dox-LP was stable up to 48 h. The long-term stability of liposomes under 4 ºC storage conditions was measured. Dox and SA-5-incorporated liposomes were stable at 4 °C for more than 90 days. Thus, stability assessment at 4 °C over 90 days is also essential to determine the aggregation behavior of liposome formulation that indicates the stability of liposome formulations during storage. Antiproliferative activity SA-5-Dox-LP suggested that the liposome was specific for HER2-positive cancer cell lines with IC_50_ values in the lower micromolar range (0.1 μM for BT-474 and A549 and 0.35 μM in Calu-3), whereas for MCF-7 cell lines that do not overexpress HER2, IC_50_ was 2 μM. The SA-5-Dox-LP had higher antiproliferative activity than the parent SA-5 compound against all the HER2 overexpressed cell lines [15]. From the IC_50_ values for human non-cancerous lung fibroblast cells (HLFs), we found SA-5-Dox-LP showed no antiproliferative effect; however, Dox-LP and free Dox showed strong antiproliferative effects indicating cytotoxic interaction. IC_50_ values of A549 cells are similar for SA-5-Dox-LP and Dox-LP, maybe because we found A549 has the least expression of HER2 protein compared to the other two HER2-positive cancer cell lines.

As a preliminary study, in vitro cellular uptake studies can indicate the evidence of a possible targeting effect. Cellular uptake studies of liposomes suggested that SA-5-Dox-LP liposomes bind to HER2 protein on the HER2 expressing cell lines BT-474 and A549 and slowly internalize by different possible mechanisms with a saturation effect, whereas in MCF-7 cell lines, Dox cellular uptake was linear, indicating non-receptor-mediated cellular uptake. Thus, the prepared liposomes exhibited a targeting effect on HER2-positive cells. There was a difference in the cellular uptake profile of BT-474 and A549. In BT-474 cells, within 30 min, there was saturation, and again, an increase in cellular uptake. We believe that the observed effect could be due to differences in HER2 expression in these cell lines [49]. BT-474 cells overexpress HER2 at a high percentage compared to A549 cells, and HER2 and EGFR undergo cellular trafficking depending on the overexpression of receptors [50]. Thus, receptor-mediated cellular uptake, direct uptake of liposomes across the cell membranes, and HER2 trafficking and expression level all will contribute to cellular uptake of liposomes. In this work, SA-5 ligand incorporation increased Dox liposome concentration at the intracellular level, which could be due to an internalization mechanism such as receptor-mediated endocytosis. To clarify targeting internalization fate, a further study that examines internalization pathways for ligand-targeted liposomes is required [51,52]. Moreover, the targeting selectivity of SA-5-Dox-LP could be seen in the results of the MCF-7 cell line, which does not overexpress HER2 and where the IC_50_ values of SA-5-Dox-LP are lower than those of Dox-LP and free Dox.

The results of cellular uptake were consistent with antiproliferative study results. Higher cellular uptake was seen for SA-5-Dox-LP formulation by the BT-474 and A549 cell lines compared to Dox-LP and free Dox (Figure 4A–C). This improvement in cellular uptake could be related to a specific interaction between the SA-5 with the HER2 receptor on the BT-474 and A549 cell lines. There could be a possibility of receptor-mediated endocytosis in the cellular uptake of SA-5-Dox-LP. However, a detailed internalization study is necessary to identify a mechanism related to cellular uptake [51,52]. In vitro drug release studies indicated that Dox was released gradually from the SA-5-Dox-LP formulation over 12 h, reaching around 80% of Dox released. The release profile of and SA-5-Dox-LP formulation was similar to the Dox-LP formulation (Figure 6A), which is supporting the cellular drug uptake result indicating the initial availability of Dox from the formulation. Steady release from the SA-5-Dox-LP formulation helps to get the drug load to the targeted tumor site of action where the Dox activity is needed to increase drug efficacy.

We have examined the anti-tumor activity potential of targeted and non-targeted different liposomal formulations in the A549 lung tumor model. The targeted SA-5-Dox-LP and SA-5 alone showed a notable reduction in tumor growth compared to other formulations free Dox, Dox-LP, lipid liposome, and vehicle control group, as seen in Figure 6B. Doxorubicin is one of the widely used first-line drug choices in many types of cancer. However, its use is mainly limited due to serious toxicity including lethal cardiotoxicity. Histology studies revealed that the lung tissue of the groups treated with the SA-5 and SA-5-Dox-LP formulations showed no histological changes as compared to other formulation groups. Furthermore, histological analysis of the liver, heart, kidney, and spleen sections showed no toxicity signs (Figure 7B). Overall, there was no toxicity sign found in samples of all the liposome formulations treated groups, which indicated that the liposome as nano-carrier could minimize the toxicity of Dox and improved in vivo safety profile. Based on our hypothesis, maximum tumor growth reduction by targeted SA-5-Dox-LP formulation could be due to a combined effect of EGFR heterodimerization inhibitory action by SA-5 and maximized the activity of functionalized Dox liposome formulation due to its EPR effect. PLA assay is used to verify the efficiency of targeted SA-5-Dox-LP formulations and SA-5 alone to inhibit EGFR heterodimerization in the NSCLC lung tumor tissue. PLA results from tumor tissue samples indicate that HER2 heterodimerization with EGFR and HER3 proteins is decreased in both the treatment group of SA-5 alone and SA-5-Dox-LP as compared to the control group (Figure 8).

To determine whether targeted ligand SA-5 peptide homes to lung tumor regions and allows targeted delivery, in vivo bio-distribution assay was carried out using fluorescently labeled lipid–peptide conjugate (SA-5-6FAM). Fluorescently labeled SA-5-6FAM peptide and Control-6-FAM peptide were injected intravenously to lung tumor mice model at the tail vein. The bio-distribution analysis revealed that high-intensity fluorescence in lung tissue compared to other organs at a 2 h time point compared to control peptide (Figure S5). The results confirmed the SA-5 peptide’s ability to specifically transmit into the targeted lung tumor site. Instead of using the liposomal formulation, we used fluorescently labeled lipid–peptide conjugate to evaluate the targeting effect of the compound to HER2 overexpressed cancer cells in vivo because nanocarriers themselves can accumulate specifically toward certain organs [53].

A similar study was done using HER2 targeted peptide-lipid derivatives for targeted delivery in breast cancer cells. Ligand peptide-grafted PEGylated liposomes (KCC-(SG)n/PEGylated liposomes) were evaluated where peptide-lipid derivatives with serine-glycine repeats were used as a spacer. The results indicated that KCC-(SG)n/PEGylated liposomes dramatically increased cellular association on HER2-positive breast cancer cells [54]. In the publication Suga et al., authors have described lipidated peptide that targets HER2 extracellular domain. The difference between this study and our study is in the spacer used and the targeting on HER2. The peptidomimetic we have designed not only binds to the HER2 extracellular domain specifically, but it also inhibits dimerization of HER2 with EGFR and HER3 [15]. Thus, the peptidomimetic we have serves a dual purpose, targeting HER2 and modulating the signal for cancer cell growth. The peptidomimetic itself has an IC_50_ value of 0.3 to 0.8 μM in HER2 positive cancer cells, and lipidated peptidomimetic has IC_50_ of around 1 μM in HER2 positive cancer cell lines. Thus, our liposome formulation has dual anti-cancer agents, a lipidized peptidomimetic and Dox-encapsulated liposome. Several other studies related to EGFR targeting by ligand-mediated liposomes have shown successful results [55]. For example, Dox-containing liposomes conjugated with MPEG-DSPE FabV fragments of cetuximab immunoliposomes showed better results for internalization within EGFR overexpressed cancer cells and regression in human breast cancer model, i.e., MDAMB-68 versus non-targeted liposomes [56]. Another study showed that anti-HER2 immunoliposomes containing Dox conjugated with fragments of trastuzumab produced better results in different HER2 overexpressing tumor xenograft models compared to non-targeted liposomes [57]. Compared to other ligands, choices of peptides for EGFR/HER2 targeting nanoparticles can offer many advantages such as lower immunogenic risk, reduced cost of development, and increased control over particle decoration density [4,58].

## 4. Materials and Methods

### 4.1. Materials

Cancer cell lines BT-474, A549, Calu-3, and MCF-7 and media RPMI1640 were purchased from ATCC (Manassas, VA, USA). BT-474 is a HER2 positive breast cancer cell line; Calu-3 and A549 are HER2 positive lung cancer cell lines. MCF-7 is a HER2 negative breast cancer cell line. Non-cancerous human lung fibroblast cells (HLFs) and fibroblast basal medium were also purchased from ATCC (Manassas, VA, USA). Doxorubicin was purchased from Astatech. Lipid Dipalmitoylphosphatidylcholine (DPPC) (Avanti Polar Lipids Inc., Birmingham, AL, USA), Cholesterol (Millipore Sigma, Burlington, MA, USA), Poly(ethylene glycol) distearoylphosphatidylethanolamine (PEG-DSPE) (Lysan Bio. Inc., Arab, AL, USA) were purchased. Sephadex G-25 was from Millipore Sigma, CellTiter-Glo was from Promega, Amicron Ultra was from EMD Millipore, Spectrum™ Spectra/Por™ 1 RC dialysis membrane tubing (6000 to 8000 Dalton Molecular Weight cutoff (MWCO)) was from Fisher Scientific (Waltham, MA, USA). Athymic nude mice (Foxn1-nude, female, 6–7 weeks old) were purchased from ENVIGO Laboratories (Indianapolis, IN, USA). Luciferase transfected A549 cells (A549 Red-FLuc NSCLC cells) were from Perkin Elmer. Proximity ligation assay was performed using the Duolink^®^ II assay kit (Sigma Aldrich, St. Louis, MO, USA). Primary antibodies for EGFR, HER2, and HER3 were from Enzo Life Sciences (Farmingdale, NY, USA).

### 4.2. Synthesis of Lipidized Peptidomimetic

Lipidized peptidomimetic-conjugate SA-5 was synthesized by solid-phase peptide synthesis using F-moc chemistry, as described in our previous report [15]. Other fluorescently labeled conjugates and controls were synthesized using a similar procedure. The conjugate was purified by high pressure liquid chromatography (HPLC) and characterized by analytical HPLC and mass spectrometry (Table S1). The molecular mass (monoisotopic mass) of the peptide-conjugate was calculated 784.53 Da, experimental m/z [M + H]^+^, 785.53.

### 4.3. Liposome Preparation

SA-5-Dox-LP consisting of dipalmitoylphosphatidylcholine (DPPC), cholesterol (CHOL), and poly(ethylene glycol) distearoylphosphatidylethanolamine (PEG-DSPE) were prepared by thin-film hydration [59]. Briefly, lipids including SA-5 (DPPC:CHOL:PEG-DSPE:SA-5 molar ratio 5.45:5.17:0.10:0.25) were dissolved in chloroform in a round-bottom flask, and the solvent was allowed to evaporate in a rotary flash evaporator [60]. The film was flushed with nitrogen for 30 min to remove any traces of chloroform.

Dox was loaded into the liposome through the remote loading method. The thin, dry lipid film was hydrated using 250 mM ammonium sulfate (pH 5.4) at 54 °C for 30 min. Then, the liposome suspension was extruded 10 times through a polycarbonate membrane with a pore size of 100 nm (Millipore, Bedford, MA, USA). Dox solution was added at pH 7.4 to create a transmembrane pH gradient (lipid to drug *w*/*w* ratio of 13:1) to the liposome solution and incubated at 37 °C for 2 h [24]. Finally, the liposome solution was passed through a Sephadex G25 gel filtration column pre-equilibrated with PBS pH 7.4 to remove the ammonium sulfate solution. Resultant liposomes were subjected to ultra-centrifugation (Amicon^®^ Ultra-15 100K device, Sigma Aldrich St. Louis, MO, USA) at 25,000 rpm, 4 °C for 20 min to remove untrapped Dox. Liposome containing only free Dox (Dox-LP) and without any Dox (Plain LP) was prepared in the same process for comparison. PEG-DSPE concentration was kept minimal to have stealth property without interfering ligand-targeting effect.

### 4.4. Characterization of Liposome

The mean particle size of liposomes was measured using a dynamic particle size analyzer (NanoBrook 900Plus Phase Analysis Light Scattering (PALS) machine). Zeta potential was measured using the NanoBrook 900Plus PALS machine in 25 °C. The morphological analysis of liposome was carried out by TEM (JEOL JEM-1400) using a negative staining method. For morphological observation, samples were placed on the grid and stained with 2% uranyl acetate and analyzed by TEM at Louisiana State University, Baton Rouge. For cryo-TEM, 4 µL sample solution was applied to a glow discharged 300 mesh carbon-coated TEM grid (EMS CF300-cu). After cold stage transfer, the samples were mounted and examined in TEM, operating at an accelerating voltage of 120 kv. The stage temperature was kept below −170 °F and images were recorded at defocus setting with Gatan US1000xp2 camera.

### 4.5. Encapsulation Efficiency Analysis

Unentrapped Dox was removed through centrifugal ultrafiltration (Amicon^®^ Ultra-15 100K device) and measured in a spectrofluorometer (excitation/emission—485/590 nm). The standard curve of Dox was measured (Supplementary Materials, Figure S2) with serial dilation: 20, 10, 5, 1.25, 0.625, 0.3125 μg/mL (Dox dissolved in PBS). The total amount of Dox is the sum of the amount of Dox loaded in a liposome (W encapsulation) and unloaded Dox (W unloaded). Encapsulation efficiency was calculated according to the formula:

EE = (W encapsulation/W total) ∗ 100%(1)

### 4.6. Stability of Liposome

To examine the stability of the liposomes, liposome solutions were kept in 4 °C storage conditions, and particle size variations were measured at different time points. SA-5-Dox-LP and Dox-LP were stored for 30, 60, and 90 days successively measured for particle size variation. In addition, in vitro serum stability was carried out according to the previously described method [61]. Briefly, human serum was centrifuged, and the supernatant was filtered with a 1 µm syringe filter to remove any suspended particles. SA-5-Dox-LP and Dox-LP solution was mixed with human serum at a 1:3 ratio and incubated at 37 °C for 0, 24, and 48 h successively. Finally, the particle size was measured at different time points (0, 24, and 48 h successively). 200 µL of the sample was taken and appropriately diluted with distilled H_2_O at different time points and analyzed in the particle size analyzer. Three replicates were analyzed for each sample type in each time point. From a single stored stability solution in a particular condition, samples were taken in different time points and measured for stability assessment.

### 4.7. Antiproliferative Activity

Antiproliferative activity of liposome formulations was determined by CellTiter-Glo^®^ assay [62].

The antiproliferative effect was examined on cancer cell lines BT-474, A549, Calu-3 (HER2-overexpressed breast and NSCLC cell lines), and MCF-7 (does not overexpress HER2) shown in Table 2. BT-474, A549, MCF-7 cells, and Calu-3 were cultured in RPMI1640 and Eagle’s Minimum Essential Medium (EMEM) media, respectively. Cells were coated on 96-well plates and incubated for 24 h (37 °C, 5% CO_2_). The cells were then treated with different concentrations of testing liposome formulations (0.01–50 µM). After 72-h incubation, cells were washed with PBS, and CellTiter-Glo^®^ detection reagent was added. Finally, luminescence measurements were performed using a Biotek plate reader. All experiments were carried out in triplicate. A dose-response plot was generated, and the IC_50_ value was calculated.

### 4.8. Time-Dependent Cellular Uptake

Cellular uptake of encapsulated Dox liposome formulations, as well as free Dox, was carried out. Breast and lung cancer cell lines such as BT-474, MCF-7, and A549 were used. Different cell lines were seeded in six-well plates and incubated at 37 °C under an atmosphere of 5% CO_2_ and 90% relative humidity. After 80% cell confluency, the medium was changed with Dox-LP, SA-5-Dox-LP formulations, and free Dox and incubated for different times (0, 0.1, 0.2, 0.3, 1, 3, 6, 12, and 24 h). Total Dox incubation was kept the same between free Dox, Dox-LP, and SA-5-Dox-LP by calculating Dox content by entrapment efficiency. Measured liposome solution and free Dox were added to cell medium in 15 mL tube prior to adding to well-plate. After the incubation time, the cells were washed with PBS solution. The cells were then solubilized by adding 0.25% Triton X-100 in PBS solution. The fluorescence intensity of the Dox was measured with a plate reader (485/590 nm). Cellular drug uptake percentage was calculated by measuring cell-related fluorescence after washing against the fluorescence measured in the feed solution.

### 4.9. Total Protein Concentration Determination

Total protein concentration was determined using Bradford assay. Breast and lung cancer cell lines such as BT-474, MCF-7, and A549 were seeded in six-well plates and incubated at 37 °C under an atmosphere of 5% CO_2_ and 90% relative humidity. After 80% cell confluency, the medium was changed with SA-5-Dox-LP formulations. The cells were washed with PBS solution in four different time points (0, 6, 12, and 24 h) and then solubilized by adding NP-40 cell lysis buffer. The protein concentrations were determined using Pierce^TM^ BCA Protein Assay Kit (Rockford, IL), and absorbance was determined using a plate reader set at 570 nm. A standard curve was plotted for each of the cell line samples to get protein concentration.

### 4.10. In Vitro Fluorescence Microscopy Studies

Fluorescence microscopy was used to compare the cellular uptake of SA-5-Dox-LP to that of Dox-LP and free Dox. First, 10,000 BT474 cells per well were cultured at 37 °C. Then the medium was removed and replaced with liposomal Dox formulations (3 μM), and the cells were incubated for 24 h. After incubation, cells were washed with PBS and then fixed by adding 500 μL of ice-cold methanol for 5–8 min and incubating the plate at −20 °C. The methanol was discarded, and immediately 500 μL of PBS was added twice to the wells to prevent cell dehydration. Finally, cells were counterstained with 4′,6-diamidino-2-phenylindole (DAPI) and mounted on slides with a coverslip. Slides were visualized under a microscope using an Olympus BX63 fluorescence microscope. Dox fluorescence was imaged with the microscope at the excitation wavelength λ 485 and emission at λ 590 nm. The fluorescence intensity of Dox was quantified using ImageJ software. Three to four different fields from each group of cells were chosen, and the intensity of Dox was normalized with the number of DAPI-stained nuclei in each field. The intensity of the image from the control sample was set to 1 arbitrary unit (a.u).

### 4.11. In Vitro Release of Dox from Liposomes Formulations

The in vitro release measurements were performed according to the dialysis method [61,63]. 1 mL of SA-5-Dox-LP, Dox-LP formulations, and free Dox were added to dialysis bags (MWCO: 6000–8000 Da) and tightly sealed. The total concentration (loading) of Dox in liposome solutions and free Dox was 862 µM. Then the bags were placed into 40 mL PBS and incubated at 37 °C for 24 h with gentle oscillating at 100 rpm. At different time points (0.5, 1, 2, 4, 8, 12, 20, and 24 h), the fluorescence intensity of the release medium was monitored using the Biotek plate reader. 500 µL of the sample was collected at each time point, and the dialysis medium was replaced with fresh PBS. The excitation and emission wavelengths were set at 485 nm and 590 nm, respectively [63]. The standard curve was measured with serial dilations of Dox solutions, and R^2^ about 0.985 was found (Supplementary Materials, Figure S2).

### 4.12. In Vivo Anti-Tumor Efficacy Evaluation

NSCLC lung cancer model is established by the following method. All animal studies were conducted according to the University guidelines and according to the approved protocol by the Institutional Animal care And User Committee (IACUC) committee, University of Louisiana Monroe, and National Institutes of Health (NIH) guidelines.

Briefly, Athymic nude mice (Foxn1-nude, female, 6–7 weeks old) were purchased from Harlan (ENVIGO) Laboratories. 4.5 × 10^6^ Luciferase transfected A549 cells (A549 Red-FLuc NSCLC cells, Perkin Elmer) in 100 µL of PBS were injected into mice via tail vein (IV) injection. Mice were monitored for one week before the intraperitoneal (IP) injection with luciferin, and bioluminescence was measured for each animal by imaging under anesthesia using an IVIS (Perkin Elmer) instrument. Two weeks after tumor cell injections (when the tumor bioluminescence reached approximately detectable range), mice were divided into six groups; vehicle (control), Dox (4 mg/kg), Dox-LP, lipid liposome, SA-5 conjugate (6 mg/kg) and SA-5-Dox-LP. All the formulations were injected via a tail vein of mice (*n* = 3) in 100 µL volume twice a week. The treatment period for each group was three weeks. Animals were imaged once a week to monitor the progression of lung cancer. After the fifth week, all animals were imaged and sacrificed. For quantification of luminescence, regions of interest (ROI) were drawn around the bioluminescent signals and quantified as photons/second (p/s). A graph of luminescence intensity vs. days of treatment in weak was plotted. Statistical analyses were carried out using an unpaired t-test to evaluate the significance between vehicle control and treatment groups. Graph Pad (GraphPad Software, San Diego, CA, USA) statistical analysis was performed by unpaired t-test. To evaluate the significance between multiple groups for each week, a two-way ANOVA followed by Tukey multiple comparison test was performed. Statistical significance was set at the level of *p* < 0.05.

### 4.13. Inhibition of EGFR Heterodimerization In Vivo

Tumor sections were deparaffinized using xylene and rehydrated with decreasing concentrations of ethanol. Antigen retrieval on the microsections of the lung tumors was done in a steaming sodium citrate buffer (10 mM, 0.05% Tween-20, pH 6.0, for 5 min). The sections were then used to analyze the inhibition of EGFR:HER2 and HER2: HER3 dimerization using proximity ligation assay, as described in the literature [64]. Briefly, sections of slides were incubated with primary antibodies (EGFR, HER2, and HER3) overnight at 4 °C and washed, and secondary antibodies with PLA probes (positive and negative) were added. Images were taken with Olympus BX63 fitted with deconvolution optics using DAPI, FITC, and Texas Red filters and processed by using CellSens dimension software.

### 4.14. Histopathology of Tissues and Assessment of Toxicity

Two days after the completion of the anti-tumor efficacy evaluation, all animals were sacrificed, and major organs were harvested for toxicity assessment. Formalin-fixed tissues were embedded in paraffin blocks and sectioned at 5 μm. Micro sections of lung tumor, heart, kidney, liver, spleen were stained with hematoxylin and eosin (H&E). The stained slides were assessed for the presence of histological changes of toxicity, including inflammation, by a certified pathologist (I.C.) blinded to the experimental conditions.

## 5. Conclusions

We have successfully developed a peptidomimetic-tagged liposome containing Dox that can effectively target HER2 overexpressed breast and lung cancer cells. The formulation was stable in serum, and in vitro release profiles indicated that SA-5-Dox-LP release was slow compared to Dox-LP and free Dox, suggesting that the formulation was stable enough to reach the targeting site. The formulation was able to reduce the NSCLC tumors in the mice model compared to the control, and no toxicity was observed in the organs of mice that are treated with the formulation. This ligand-mediated liposome may be used to enhance the therapeutic efficiency of Dox in HER2-positive or EGFR dimers that are important in breast and NSCLC.

## Figures and Tables

**Table 1:** Particle size, zeta potential, polydispersity index, and encapsulation efficiency of different liposome formulations. Values are from triplicate measurements. Each data represents mean ± S.D.

Formulation	Particle Size (nm)	Zeta Potential (mV)	PDI	Encapsulation Efficiency
SA-5-Dox-LP	107.19 ± 2.90 nm	−13.38 ± 1.50	0.17 ± 0.05	86.73 ± 4.63%
Dox-LP	105.23 ± 3.20 nm	−4.18 ± 0.70	0.15± 0.05	82.60 ± 1.74%
Plain LP	98.45 ± 1.74 nm	−2.32 ± 0.26	0.16± 0.03	-

**Table 2:** Antiproliferative activity of liposome formulations in cancer cells BT-474 (HER2+ breast cancer cells), Calu-3 (HER2 + lung cancer cells), A549 (HER2 + lung cancer cells), breast cancer cells that do not overexpress HER2 receptors (MCF-7), and non-cancerous human lung fibroblast cells (HLFs). The activity was represented as IC_50_ values in *µ*M. Each data represents mean ± S.D.

	IC_50_ in μM (72 h)				
Formulation	BT-474	Calu-3	A549	MCF-7	HLFs
SA-5-Dox-LP	0.10 ± 0.10	0.35 ± 0.07	0.10 ± 0.02	2.20 ± 0.30	>50
Dox-LP	1.80 ± 0.70	0.50 ± 0.10	0.11 ± 0.07	0.10 ± 0.70	7.84 ± 0.41
Free Dox	0.08 ± 0.05	0.04 ± 0.02	0.03 ± 0.04	0.04 ± 0.10	0.15 ± 0.04
Plain LP	>50	>50	>50	>50	>50

## Data Availability

The data presented in this study are available in Supplementary Materials.

## Supplementary Materials

The following are available online at https://www.mdpi.com/1424-8247/14/3/221/s1, Analytical data on peptidomimetic conjugates, fluorescently labeled peptidomimetics and details of parameters of particle size analysis and standard curves for cellular uptake, TEM images of the liposome, in vivo imaging of mice with lung cancer (file type, PDF).
