# Supplementary material for: Lipidated Peptidomimetic Ligand-Functionalized HER2 Targeted Liposome as Nano-Carrier Designed for Doxorubicin Delivery in Cancer Therapy
Source: Pharmaceuticals (Basel). 2021 Mar 6;14(3):221. doi: 10.3390/ph14030221 (PMC8002094; doi:10.3390/ph14030221)
Supplement: Supplementary file 1 [file pharmaceuticals-14-00221-s001.pdf]

Lipidated Peptidomimetic Ligand Functionalized HER2 targeted Liposome as Nano-carrier Designed for Doxorubicin Delivery in Cancer Therapy.

Himgauri Naik[1], Jafrin Jobayer Sonju[1**], Sitanshu Singh[1], Ioulia Chatzistamou[2], Leeza Shrestha[1], Ted Gauthier[3], and Seetharama Jois[1*]**

Supporting Information

Table S1. The sequence of peptides and conjugates described in this study, along with analytical data*.

Compound	Sequence	Experimental Molecular Weight	Calculated Molecular Weight	Purity
SA-5	Stearic acid-Arg-(*S*)Anapa-Phe-OH	785.53	785.06	>95.
SA-5-6FAM	Stearic acid-Gly-Arg-(*S*)Anapa-Phe-Asp-Gly-Gly-Gly-Lys-OH (FAM Lys side chain)	1614.94	1614.83	>90
SA-Control-6FAM	SA-Gly-Arg- Ala-Ala-Leu-Gly-Gly-Gly--Lys-OH [FAM-Lys.Side Chain]	1410.91	1410.65	>90

*also reported in our earlier studies Naik et al. Bioorg Med Chem Lett. 2018 Dec 1;28(22):3506-3513.

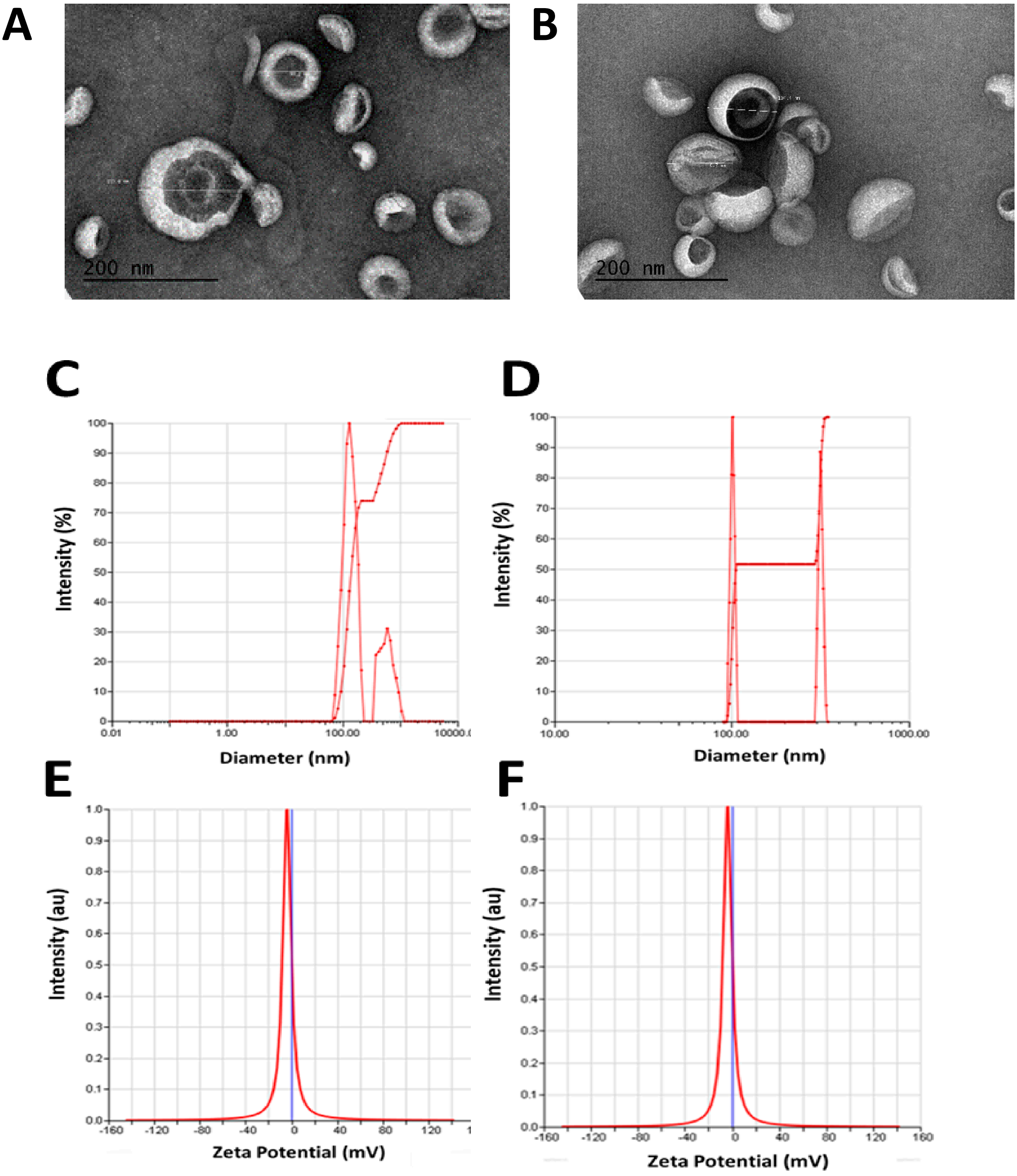

Figure S1. TEM images of **A)** Dox-LP and **B)** Plain LP in 200 nm magnification. The size distribution of **C)** Dox-LP and **D)** Plain LP; zeta potential graph of **E)** Dox-LP and **F)** Plain LP. Details of sample preparation and analysis are provided in the text.

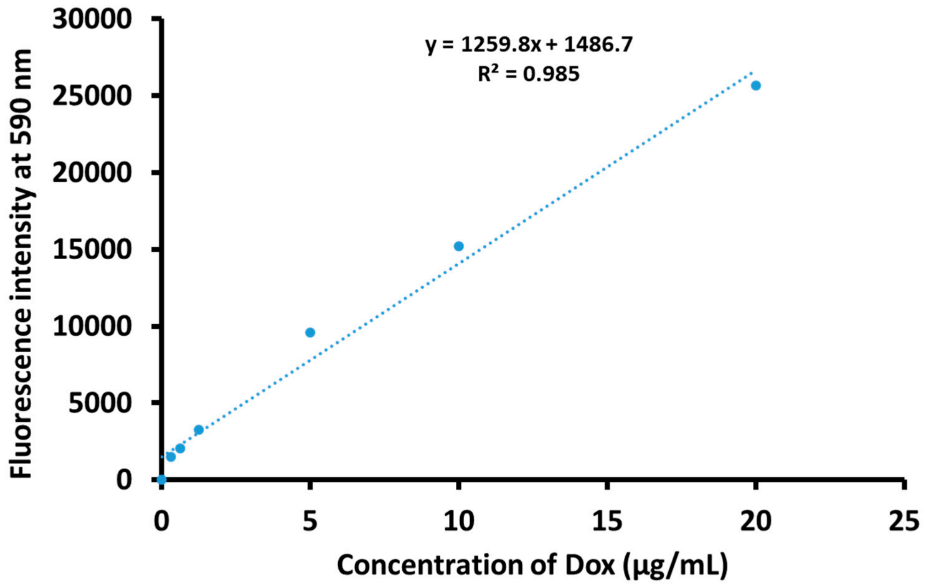

Figure S2. Standard curve of Dox in PBS obtained by serial concentrations: 20, 10, 5, 1.25, 0.625, 0.3125 µg/mL.

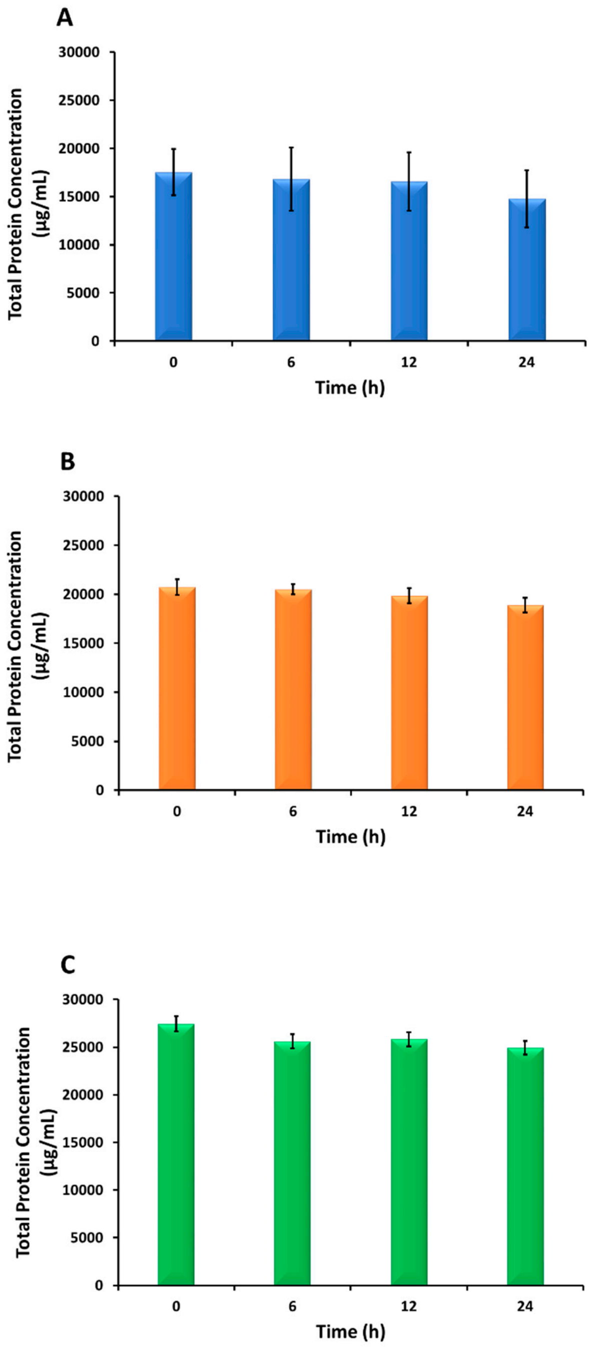

Figure S3. Total protein concentration in cells treated with SA-5-Dox-LP in A) A549, B) BT-474 (HER2+ve), and C) MCF-7 (HER2-ve) cell lines at 0 to 24 h. Data represented as mean ± standard deviation.

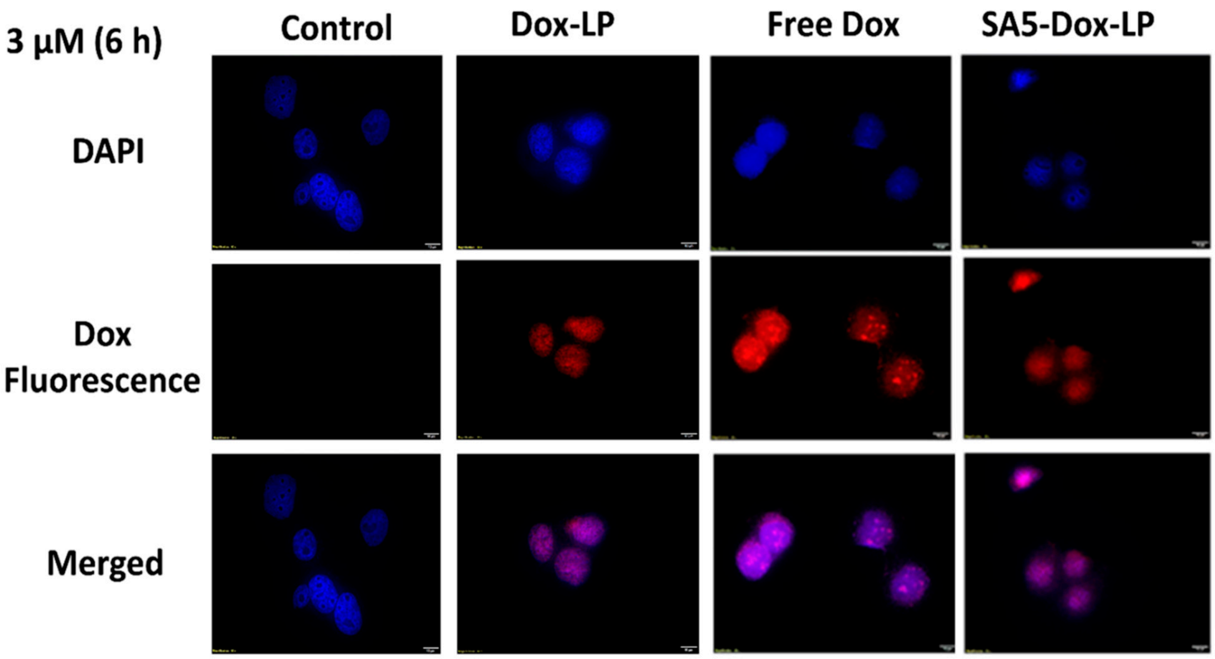

Figure S4. Fluorescence images of cellular uptake studies of SA-5-Dox-LP, Dox-LP, and free Dox in MCF-7 cells. Cells were incubated with different formulations for 6 h and fixed after washing. Fluorescence from doxorubicin was imaged along with nuclear stain DAPI. The figure shows doxorubicin fluorescence and DAPI fluorescence, and merged images. The merged image indicates that in 6 h, SA-5-Dox-LP formulation was taken up by cells and entered the nucleus. In the case of free Dox, there was less number of cells in 24 h as Dox has a cytotoxic effect on cells. Clusters of cells are shown. Magnification 60×.

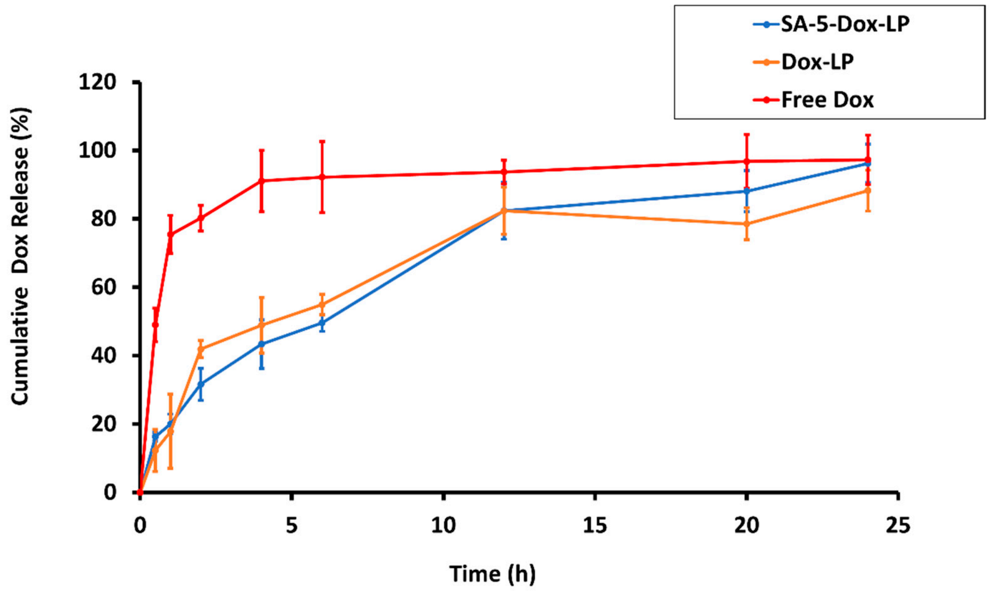

Figure S5. *In vitro* drug release from SA-5-Dox-LP and Dox-LP at pH 7.5 without the addition of tween. Results were from triplicate experiments. Dox release was monitored by measuring the fluorescence of doxorubicin and expressed as a percentage calculated as described in the text. LP-liposome. Each data represents mean ± S.D

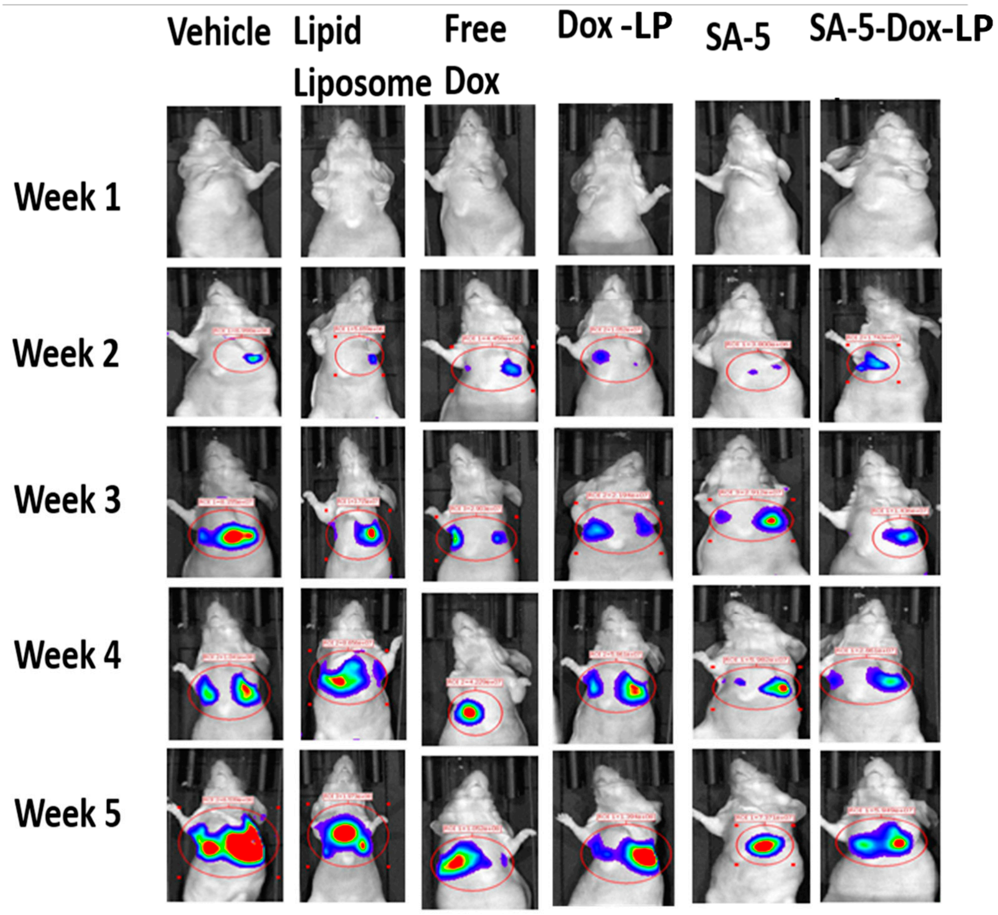

Figure S6: In vivo imaging of different formulation groups from week1 to week 5 study timeline. SA-5 and SA-5-Dox-LP formulation groups showed a reduction in tumor size compared to the vehicle and lipid liposome control group.

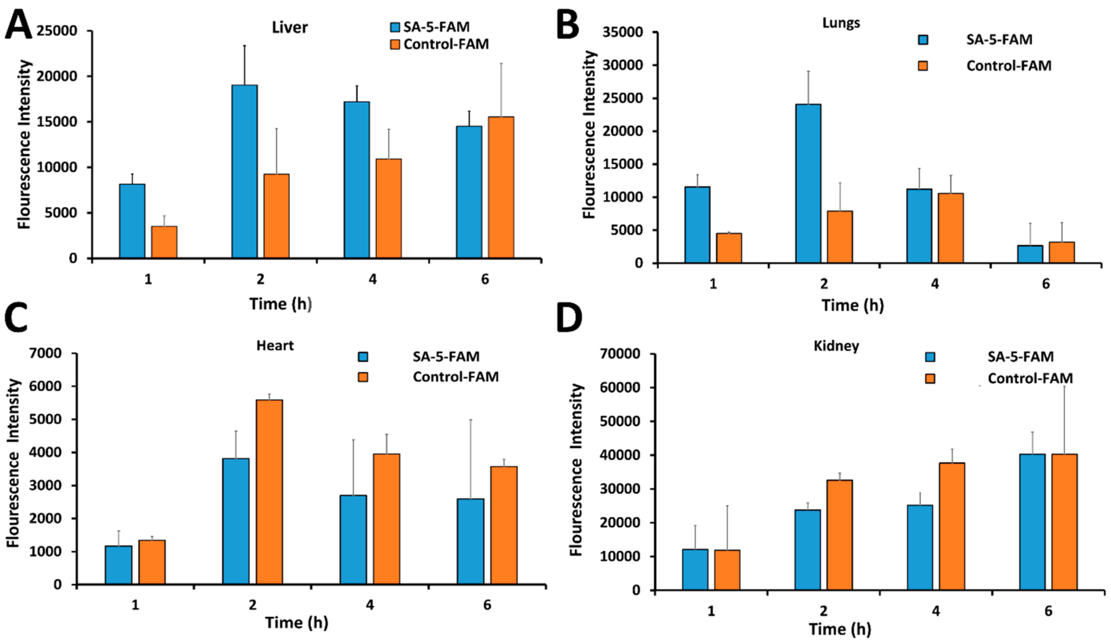

Figure S7: In vivo biodistribution of SA-5-6 FAM and Control-6FAM at different time points 1, 2, 4, and 6 h in NSCLC lung cancer model. A) Liver, B) Lungs, C) Heart, D) Kidney.

Bio-distribution Study

All animal studies were conducted according to the University guidelines and according to the approved protocol by the IACUC committee, University of Louisiana Monroe, and NIH guidelines. Athymic nude mice (Foxn1-nude, female, 6–7 weeks old) purchased from ENVIGO. 4.5 × 10^6 Luciferase transfected A549 cells (A549 Red-FLuc NSCLC cells, Perkin Elmer) in 100 µl of PBS were injected into mice via tail vein (IV) injection. Mice were monitored for one week before the intraperitoneal (IP) injection with luciferin, and bioluminescence was measured for each animal by imaging under anesthesia using an IVIS (Perkin Elmer) instrument. To examine the targeted delivery potential of SA-5 peptide ligand in vivo, we used fluorescent-labeled peptides SA-5-6FAM and Control-6FAM as a control peptide in the biodistribution study (**Table S1**). Fluorescently labeled peptides stock solutions were prepared in DMSO and diluted in PBS to achieve less than 1% DMSO in the final solution. The final concentration of the fluorescently labeled peptide injected was 6 mg/kg (100 µL volume). Peptides were injected into the tail vein via IV. At different times points of 1, 2, 4, and 6 h, three lung tumor-bearing mice per group were sacrificed and harvested major organs lung tumor, heart, liver, kidney, and spleen. All organs were homogenized in PBS

solution. Further homogenized sample subjected to centrifugation for 10 min at 10,000 rpm. Finally, the supernatant was collected, and fluorescence intensity measured using a Biotek fluorescence plate reader at λex 485 nm and λem 530 nm.

In Vivo Biodistribution of SA-5FAM

It was important to determine whether targeted ligand SA-5 peptide homes to lung tumor regions and allows targeted delivery. Here instead of using the liposomal formulation, we used fluorescently labeled lipid peptide conjugate to evaluate the targeting effect of the compound to HER2 overexpressed cancer cells in vivo. Compound 5 is known to bind to the HER2 protein, and it was conjugated with stearic acid and fluorescent label 6FAM. We used fluorescently labeled SA-5-6FAM peptide and Control-6-FAM peptide and then injected intravenously to lung tumor mice model at the tail vein. Fluorescence from the tissue compared to the control indicated that SA-5 conjugate is distributed in different organs in the mice, but fluorescence was of high intensity in lung tissue compared to other organs at 2 h time point compared to control peptide (**Fig. S7**).
